# Evaluation of Berberine as an Adjunct to TB Treatment

**DOI:** 10.3389/fimmu.2021.656419

**Published:** 2021-10-20

**Authors:** Mumin Ozturk, Julius E. Chia, Rudranil Hazra, Mohd Saqib, Rebeng A. Maine, Reto Guler, Harukazu Suzuki, Bibhuti B. Mishra, Frank Brombacher, Suraj P. Parihar

**Affiliations:** ^1^ International Centre for Genetic Engineering and Biotechnology (ICGEB), Cape Town, South Africa; ^2^ Division of Immunology and South African Medical Research Council (SAMRC) Immunology of Infectious Diseases, Institute of Infectious Diseases and Molecular Medicine (IDM), Department of Pathology, Faculty of Health Sciences, University of Cape Town, Cape Town, South Africa; ^3^ Wellcome Centre for Infectious Diseases Research in Africa (CIDRI-Africa) and Institute of Infectious Diseases and Molecular Medicine (IDM), Faculty of Health Sciences, University of Cape Town, Cape Town, South Africa; ^4^ Division of Medical Microbiology, Institute of Infectious Diseases and Molecular Medicine (IDM), Department of Pathology, Faculty of Health Sciences, University of Cape Town, Cape Town, South Africa; ^5^ Department of Immunology and Microbial Disease, Albany Medical College, Albany, NY, United States; ^6^ Department of Molecular and Cell Biology, University of Cape Town, Cape Town, South Africa; ^7^ RIKEN Center for Integrative Medical Sciences, Yokohama, Japan

**Keywords:** tuberculosis, host-directed therapy, berberine, anti-inflammatory, C57BL/6 and C3Heb/FeJ Kramnik mice

## Abstract

Tuberculosis (TB) is the global health problem with the second highest number of deaths from a communicable disease after COVID-19. Although TB is curable, poor health infrastructure, long and grueling TB treatments have led to the spread of TB pandemic with alarmingly increasing multidrug-resistant (MDR)-TB prevalence. Alternative host modulating therapies can be employed to improve TB drug efficacies or dampen the exaggerated inflammatory responses to improve lung function. Here, we investigated the adjunct therapy of natural immune-modulatory compound berberine in C57BL/6 mouse model of pulmonary TB. Berberine treatment did not affect *Mtb* growth in axenic cultures; however, it showed increased bacterial killing in primary murine bone marrow-derived macrophages and human monocyte-derived macrophages. *Ad libitum* berberine administration was beneficial to the host in combination with rifampicin and isoniazid. Berberine adjunctive treatment resulted in decreased lung pathology with no additive or synergistic effects on bacterial burdens in mice. Lung immune cell flow cytometry analysis showed that adjunctive berberine treatment decreased neutrophil, CD11b^+^ dendritic cell and recruited interstitial macrophage numbers. Late onset of adjunctive berberine treatment resulted in a similar phenotype with consistently reduced numbers of neutrophils both in lungs and the spleen. Together, our results suggest that berberine can be supplemented as an immunomodulatory agent depending on the disease stage and inflammatory status of the host.

## Introduction

Tuberculosis (TB) is currently the second deadliest infectious disease worldwide caused by a single pathogen with an estimated 10 million cases and 1.4 million deaths reported in 2019 (1). Although it has been previously stated that between one-third to one-fourth of the world population has latent tuberculosis infection (LTBI) according to the epidemiological modeling studies, this notion has been challenged recently (2, 3). There is still an alarmingly large reservoir of active TB cases due to a 5-10% risk of LTBI progression (4). In addition to ongoing transmission in communities, the treatment for TB is long and complex with considerable side effects. Poor compliance, inadequate health infrastructure for drug monitoring has resulted in the increasing prevalence of multidrug (MDR) and extensively drug-resistant (XDR) TB. A recent study forecasted that by 2040, 32.5% of incident TB cases in Russia and 12.4% in India will be MDR-TB even though acquired drug resistance rates have been dropping (5). TB also impinges on adults during their economically productive life period furthermore, it customarily affects impoverished and socially disadvantaged communities to a greater extent. It is estimated that TB will cost the global economy $983 billion between 2015-2030 (6). Therefore, the development of novel treatment options with a lower toxicity profile that can synergize with the existing first-line and second-line antibiotics to decrease treatment duration is the focus of attention. There has been substantial progress in the anti-mycobacterial field with the recent approvals of bedaquiline, delamanid and pretomanid. Besides antimycobacterials; adjunct host-directed therapies (HDT) are also in the spotlight as alternative approaches to exploit host-pathogen interplay. Improving bacterial killing mechanisms, reinforcing immune and memory responses, disrupting TB granuloma structure and balancing inflammatory responses can be targeted for HDT (7, 8). One of the hallmarks of pulmonary TB is inadequately regulated inflammatory responses which exacerbate tissue damage, necrosis and eventual lung cavitation (9). An immunomodulatory HDT that fine-balances the host inflammatory pathway can dampen excess host inflammatory response and long term lung damage.

Berberine is the bioactive ingredient extracted from roots, barks, rhizomes of medicinal plant families Berberidaceae (barberry), Ranunculaceae (goldenseal), Rutaceae (cork tree) and Annonaceae (African whitewood). Berberine is an isoquinoline alkaloid with antimicrobial, antidiabetic, anti-tumor and anti-inflammatory properties (10–14). As a Chinese and Native American traditional medicine, it has been used for the treatment of gastroenteritis and dysentery (15). The diverse pleiotropic actions of berberine are mainly ascribed to its immunomodulatory properties through inhibition of nuclear factor kappa B (NF-κB), mitogen-activated protein kinase (MAPK) signaling pathways and inhibition of pro-inflammatory cytokine production (16). Berberine can also affect cell proliferation, cell death and inhibit prolonged activation of immune cells. In the experimental autoimmune neuritis model, berberine treatment ameliorated the development of the autoimmune disease by inhibiting CD4^+^ T cell proliferation (17). In the collagen-induced arthritis model, berberine treatment induced apoptosis of IL-12 producing mature dendritic cells in spleen and lymph nodes of mice that can result in subsequent restriction of chronic activation of T cells (18). In dextran sulfate sodium (DSS)-induced colitis model, berberine induced apoptosis of colonic macrophages and decreased pro-inflammatory cytokine production from colonic macrophages and colon epithelial cells (19). In trinitrobenzene sulfonic acid (TNBS)-induced colitis model, berberine treatment shifted macrophages into tissue repair and remodelling associated M2 phenotype rather than tissue destructive, pro-inflammatory M1 phenotype (20).

The immunomodulatory mechanisms of berberine in diverse inflammatory disease models prompted us to investigate the potential of berberine as an adjunct therapy in C57BL/6 murine model of tuberculosis. In the present study, we sought out synergistic effects of berberine with first-line antimycobacterials rifampicin and isoniazid in well-established murine models. Our results suggest that berberine treatment decreases tissue pathology without any additive or synergistic effects on the bacterial burden. The decrease in tissue pathology correlates with a decreasing number of inflammatory neutrophils, recruited macrophages and CD11b^+^ dendritic cells in the C57BL/6 model when the treatment started at earlier stages of infection. However; the effect on decreased inflammatory cells was not observed once berberine treatment started at later stages of C57BL/6 *Mtb* infection. Our results suggest that berberine adjunctive treatment can exert its beneficial effects depending on the inflammatory stage of the host during tuberculosis.

## Materials and Methods

### Mice

8-10 weeks old C57BL/6 mice were kept under specific-pathogen-free conditions in a biosafety level 3 containment facility individually ventilated cages (5 mice per cage) with filter tops (type 2 long), as well as dried wood shavings and shredded filter paper as floor coverings. The temperature range was set at 22–24°C and 12h dark–12h light cycles. All experiments were performed in accordance with the South African National Guidelines and the University of Cape Town of practice for laboratory animal procedures. The protocol was approved by the Animal Ethics Committee (AEC Permit Number: 015/040), Faculty of Health Sciences, University of Cape Town, Cape Town, South Africa. Similarly, C3HeB/FeJ (Kramnik) mice were kept under specific-pathogen-free conditions in individually ventilated cages at the animal resource facility at Albany Medical College, Albany, New York. All experiments were performed in accordance with the IACUC guidelines at the Albany Medical College, New York.

### *Mtb* Growth Assay

Two-fold diluted concentrations of berberine (Sigma Aldrich, B3251) were screened for their anti-mycobacterial activity in 96-well, black, clear-bottom microplates (Greiner Bio-One, Germany), as previously reported (21). Single-cell suspension of constitutively GFP expressing H37Rv Mtb strain from frozen glycerol (15%) stock with a working concentration of 1x10^6^ colony-forming unit (CFU)/mL was prepared in Middlebrook 7H9 (Difco™, BD Biosciences) supplemented with 25 µg/mL kanamycin (Sigma Aldrich), 10% Middlebrook Oleic Acid-Albumin-Dextrose-Catalase (OADC) (v/v), 0.05% Tween 80 (w/v) and 0.2% glycerol (v/v). 100 µl of H37Rv-GFP was added to each experimental well followed by 100 µl 2X concentrated of berberine prepared in 7H9 broth as described above to test the 3.9 µM to 250 µM concentration range. Fluorescence (485ex/520em nm) was measured at days 0, 4, 6, 8, 10 and 12, with a BMG Labtech Omega Plate Reader (Germany).

### Bone Marrow-Derived Macrophage (BMDM) and Monocyte-Derived Macrophage (MDM) Generation and Intracellular CFU Assay

Bone marrow-derived macrophages were generated from 8-12 weeks old C57BL/6 mice. Generation of BMDM was performed as described previously (22). BMDM were seeded in tissue culture-treated 96-well flat-bottom plates (Costar^®^, Corning) at a concentration of 1x10^6^ cells/ml. The cells were treated with 30µM berberine overnight, followed by H37Rv infection. In other sets of experiments, BMDM were rested overnight and infected with H37Rv. After 4 hours of infection, media was supplemented with berberine or vehicle (DMSO final concentration 0.1%).

Monocyte-derived macrophages were generated from Leukopak obtained from Western Province Blood Service. Briefly, Leukopak was diluted 1:1 with phosphate buffered saline (PBS) containing 2% fetal bovine serum (Gibco, ThermoFisher) and centrifuged at 500g for 25 minutes with brakes off in Leucosep (Greiner Bio-one) tubes with Histopaque 1077 (Sigma Aldrich). The buffy coat is removed by Pasteur pipette and washed twice with PBS+2%FBS at 120g to remove platelets. Peripheral blood mononuclear cells were counted and subjected to CD14^+^ positive selection (Miltenyi) according to the manufacturer’s instructions. Isolated monocytes were seeded in 60 mm Nunc cell culture dishes (ThermoFisher) at a concentration of 1x10^6^ cells/ml in RPMI 1640 media (Gibco, ThermoFisher) supplemented with 10% human AB serum (Sigma-Aldrich), 50 U/ml penicillin G (ThermoFisher), 50 µg/ml streptomycin (ThermoFisher) and 50 ng/ml recombinant human M-CSF (Peprotech) for 7 days. MDM were harvested by 20 minutes of incubation in Accutase^®^ (Sigma-Aldrich) solution. MDM were seeded in tissue culture-treated 96-well flat-bottom plates (Costar^®^, Corning) at a concentration of 1x10^6^ cells/ml without the antibiotics. The cells were then treated with 30µM berberine overnight, followed by H37Rv infection.

BMDM and MDM were infected with a multiplicity of infection (MOI) 1. At 4 hours, 2 days and 5 days post-infection, the cells were washed once with sterile PBS and lysed in 0.1% Triton X-100. The cell lysates were diluted 10- and 100-fold and plated in Middlebrook 7H10 (Difco™, BD Biosciences). 7H10 plates incubated at 37°C for 14 days and colonies are counted under Nikon SMZ800N stereomicroscope.

### BMDM Activation and Reactive Oxygen Species (ROS) Assay

1x10^6^ BMDM were infected with H37Rv for 2 days in 12 well plates. The media was removed, and cells were washed once with PBS and incubated for 10 min in 0.5 mg/ml lidocaine and 10 mM EDTA in PBS at 37°C. Cells were lifted by pipetting and washed with PBS. The flow cytometry staining protocol mentioned below was followed. 575V Viability Dye, CD11b-PerCPCy5.5 (Clone M1/70), F4/80-PeCy7 (Clone BM8), MHCII-AlexaFluor700 (Clone M5/114.15.2), CD80-BV421 (Clone 16-10A1) were used for staining. BMDM were seeded on 96 well black/clear bottom plates (ThermoFisher) and infected with H37Rv for two days. Infected BMDM were incubated with 5 µM CellROX Green Reagent (ThermoFisher) according to manufacturer’s instruction. Uninfected BMDM were used as blank wells. The fluorescence was measured (485 nm excitation/525 nm emission) on Spectramax iD3 multi-mode reader (Molecular Devices).

### Western Blot Analysis

3x10^6^ BMDM were seeded in 6-well plates and pretreated with berberine (30 µM) or vehicle (DMSO 0.1%) overnight. BMDM were infected H37Rv for 30, 60 and 120 minutes and washed with cold PBS before lysing with RIPA buffer (150 mM NaCl, 50 mM Tris-HCl, 1% Nonidet P-40, 0.5% sodium deoxycholate, 0.1% SDS) including cOmplete Protease inhibitor and PhosSTOP phosphatase inhibitor cocktail (Roche). Cell lysate protein content was determined using the BCA Protein Assay Kit (ThermoFisher). 30 µg of protein was loaded to 10% resolving acrylamide gel and wet WB was performed onto a nitrocellulose membrane. The membrane was probed with either SAPK/JNK Antibody, Phospho-SAPK/JNK (Thr183/Tyr185) (G9), NFκB p65 (D14E12), Phospho-NFκB p65 (Ser536) (93H1) (Cell Signaling Technology) or GAPDH (Santa Cruz Biotechnology) primary antibodies and either with goat anti-rabbit IgG H&L (HRP) pre-absorbed or goat anti-mouse IgG H&L (HRP) pre-absorbed (Abcam) secondary antibodies. Immunoblots were developed using the KPL LumiGLO ^®^ Reserve Chemiluminescent Substrate Kit (SeraCare) on the iBright FL1000 Imaging System (Thermo Fisher).

### Aerosol Infection and Treatment of Mice

*Mycobacterium tuberculosis* H37Rv was grown in Middlebrook 7H9 broth as described previously (23). For infection of the C3HeB/FeJ mice, *Mtb* Erdman strain was used. Prior to infection, stock solutions of *Mtb* were thawed, washed once with phosphate-buffered saline and inoculum was prepared in sterile saline containing 0.05% Tween 80. Aerosol infection was performed using an inhalation exposure system (model A4224, Glas-Col). To infect mice with a low dose of 100 CFU/lung, animals were exposed for 40 min to an aerosol generated by nebulizing approximately 6 ml of a suspension containing 2.4x10^7^ live bacteria. The infection dose was checked at one day post-infection by determining the bacterial load in the lungs of four infected mice. One-week post-infection, four groups of mice were left untreated, treated with berberine (1 mg/ml), isoniazid/rifampicin (both 0.1 mg/ml), isoniazid/rifampicin/berberine (both 0.1mg/ml and 1 mg/ml, respectively) in drinking water. Berberine concentration was determined according to previously published reports (24). Drinking water was supplemented with 1% glucose in all four groups due to decreased water intake in the isoniazid/rifampicin/berberine triple-drug group. Glucose supplemented drug treatment groups showed similar water intake. Drinking water was changed twice a week and volume was measured to estimate the average drug intake. On average, berberine-treated groups received 5.5 mg berberine per day and isoniazid/rifampicin groups received 0.6 mg rifampicin/isoniazid (RIF/INH). At the experimental endpoint, mice were euthanized with halothane and cardiac puncture was performed for confirmation of death by exsanguination. The blood was left at room temperature for 30 min to clot and centrifuged at 1500g for 10 min at 4°C. The serum was collected to measure liver ALT and AST enzyme levels at National Health Laboratory Services (NHLS) diagnostic laboratory.

### Determination of Mycobacterial Loads and Lung Histopathology

Mycobacterial loads in the lungs and spleen of *Mtb*-infected mice were determined as previously described (25). The right superior lobes were fixed with 10% neutral-buffered formalin, and tissue was processed with the Leica TP 1020 benchtop processor for 24 h and embedded in paraffin wax. Four 3 µm thick sections with 30 µm distance apart were cut in Leica Sliding Microtome 2000R, deparaffinized and subsequently stained with the hematoxylin & eosin (H&E) stain. The lung images were acquired in Nikon 90i Eclipse widefield microscope and free alveolar space was quantified using NIS elements (Nikon Corporation, Japan). Briefly, the images were converted to binary, and H&E positive area was measured. Fill holes function of NIS elements was employed to measure the complete lung area including the alveolar spaces. Free alveolar spaces were calculated by subtracting H&E positive area from the complete lung area and presented as a percentage to the complete lung area.

### Lung and Spleen Immune Cell Populations

Single-cell suspensions of the left lobes of the lung were prepared as previously described (26). For late-onset berberine treatment experiments, half of the spleen was mechanically digested sequentially through 100 µm and 70 µm cell strainers (SPL Life Sciences). The cells were washed once with media (DMEM+10%FCS) and red blood cells were lysed by ACK lysis buffer (150 mM NaCl, 10 mM KHCO_3_ and 0.1mM Na_2_EDTA) for 5 min incubation at room temperature. The cells were washed once with media again and counted with CytoSMART (Corning) automated cell counter. Briefly, 1x10^6^ cells were washed once with PBS and stained with dead cell marker (575V Viability Dye, BD Biosciences) for 15 min at room temperature. The staining was later quenched and washed with 0.5%BSA in PBS and cells were then subjected to staining for B cells (CD3^-^CD19^+^), CD4 T cells (CD19^-^CD3^+^CD4^+^), CD8 T cells (CD19^-^CD3^+^CD8^+^), alveolar macrophages (SiglecF^+^CD11c^+^CD64^+^), neutrophils (CD11b^+^Ly6G^+^), CD11b^+^ dendritic cells (CD64^-^CD11b^+^CD11c^+^MHCII^+^) and inflammatory macrophages (CD64^+^CD11c^-^CD11b^+^SiglecF^-^) and monocyte-derived DCs (CD64^+^CD11c^+^CD11b^+^) in the presence of 10% heat-inactivated rat serum and 10% FcγR blocker for 30min on ice. Similarly, neutrophils (CD11b^+^Ly6C^+^Ly6G^+^), inflammatory monocytes (CD11b^+^Ly6C^+^Ly6G^-^) and T cells (CD11b^-^CD3e^+^Ly6C^-^Ly6G^-^) were identified in the lungs of C3HeB/FeJ mice. Spleen myeloid populations were identified as neutrophils (CD11b^+^Ly6G^+^), monocytes (CD11b^+^Gr-1^+^CD11c^-^Ly6G^-^), CD169^+^ macrophages (CD11b^+^CD169^+^CD11c^-^), red pulp macrophages (F4/80^+^CD11b^low^CD169^-^CD11c^-^), CD11b DC (CD11c^+^MHCII^+^CD11b^+^CD8^-^) and CD8 DC(CD11c^+^MHCII^+^CD8^+^CD11b^-^). Antibodies used for flow cytometry analysis were as follows: CD64-PeCy7 (Clone X54-5/7.1), Ly6C-PerCPCy5.5 (Clone AL-21), CD11b-V450 (Clone M1/70), MHCII-APC (Clone M5/114.15.2), CD11c-A700 (Clone HL3), SiglecF-APCCy7 (Clone E5-2440), Ly6G-FITC (Clone 1A8), CD4-BV510 (Clone RM4-5), CD3-A700 (Clone 500A2), CD19-PerCPCy5.5 (Clone 1D3) and CD8-APC (Clone 53-6.7), Gr-1 Biotin (Clone RB6-8C5), CD169 APC-eFluor780 (Clone Ser-4) and Streptavidin-PECF594 purchased from BD Biosciences and eBioscience. Cells were washed then fixed in 2% paraformaldehyde overnight and acquired by BD LSRII (BD Pharmingen) and analysed by FlowJo V9 (TreeStar, US). Marker positive stained cells are calculated as a percentage of live cells and later these percentages are back-calculated to cell counts obtained from Trypan Blue exclusion method and finally back-calculated by multiplying the ratio of total lung weight to the left lobe weight.

### Analysis of Cytokines in Tissue Homogenates and Culture Supernatants

The cell-free lung and spleen homogenate was spun at 3000g for 5 min and stored at -80⁰C until ELISA analysis, samples were thawed and double filtration (0.2 µM) was performed before transporting the supernatant from biosafety level 3 facility. Lung homogenates were analysed for the IFNγ, IL-12p40, GM-CSF (BD Biosciences), IFN-β, IL-10 (BioLegend), IL-1β, CXCL10, and CCL3 (R&D Systems) by ELISA according to manufacturers’ instructions. Similarly, supernatants from *Mtb*-infected macrophages were collected and stored at -80°C until further analysis. After thawing, the supernatants were filtered through Corning FiltrEX 96-well low protein binding filter plates and the supernatant was transported out of BSL3. Nitrite concentrations were measured by Griess assay. IL-1α (R&D Systems), IL-6 (BD Biosciences) and TNF (Biolegend) ELISA were performed according to the manufacturers’ instructions.

### Gene Expression in Total Lung Tissue

Lung middle lobes were washed with cold PBS and immersed in RNAprotect Tissue reagent (Qiagen) overnight at 4°C and the next day transferred to -80oC for long-term storage. On the day of RNA purification, the sample was thawed, transferred in RLT buffer and homogenized by sonification. RNA extraction was performed by RNeasy Mini Kit (Qiagen), total RNA was transcribed into cDNA using High-Capacity cDNA Reverse Transcription kit (ThermoFisher) according to the manufacturer’s instructions. Real-time qPCR was performed with SYBR Green PCR Master Mix (ThermoFisher) in QuantStudio 7 Pro Real-Time PCR System (ThermoFisher). Quantitative expression analyses of Ifngr1and Csf2ra were normalized against the housekeeping gene Hprt. qPCR primers as follows; Hprt forward 5’- GTTGGATATGCCCTTGAC-3’, reverse 5’- AGGACTAGAACACCTGCT-3’; Ifngr1 forward 5’-TACAGGTAAAGGTGTATTCGGGT-3’, reverse 5’- ACCGTGCATAGTCAGA TTCTTTT-3’; Csf2ra forward 5’- CCTGCTCTTCTCCACGCTAC-3’, reverse 5’- CAACCGAAGGGCGAGACT-3’.

### Statistics

Data are represented as mean values ± SEM. Statistical analysis was performed using one-way ANOVA with Tukey *post-hoc* test if the data points are normally distributed. The normality is checked through the Shapiro-Wilk normality test and the Anderson Darling test. If the data did not fit into Gaussian distribution Kruskal-Wallis test was performed. Statistical differences in all groups are shown as significant *, P ≤ 0.05; **, P ≤ 0.01; ***, P ≤ 0.001. All the data were plotted using the GraphPad Prism 8 software.

## Results

### Berberine Decreased Growth of *Mycobacterium tuberculosis* in Primary Macrophages and Modulated Responses to the Infection

**Figure 1:**
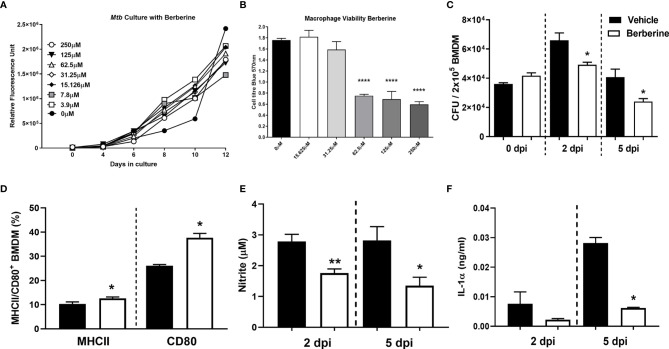
Cytotoxic effect of berberine on *Mycobacterium tuberculosis* (*Mtb*) and macrophages. **(A)** *Mtb* was cultured in 7H9 culture broth at the indicated concentrations of berberine to determine the mycobactericidal activity. **(B)** C57BL/6 murine BMDMs were treated with indicated concentrations of berberine for 3 days to measure cellular viability using Cell Titer Blue assay. **(C)** Murine macrophages were pre-treated with berberine (30 µM) overnight. Cells were then infected with *Mtb* (MOI=1) to determine the growth in a time-dependent manner. **(D)** BMDM were infected with *Mtb* for two days and stained for MHCII and CD80 antibodies to measure macrophage activation in the presence of berberine. **(E, F)** Cell culture supernatants were analysed for nitrite by Griess Reagent Assay and IL-1α by ELISA. Data represented as mean ± SEM of triplicates and representative of two experiments, analysed by one-way ANOVA with Tukey *post-hoc* test **(A, B)** or two-tailed unpaired Student’s t-test **(C, D)** or Mann-Whitney test **(E, F)** defining differences in all groups as significant *P ≤ 0.05; **P ≤ 0.01; ****P ≤ 0.0001.

**Figure 2:**
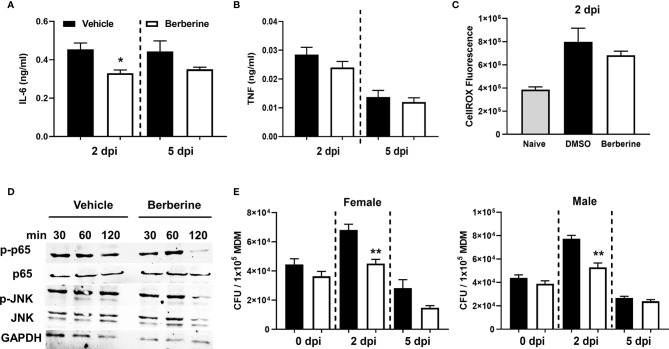
Anti-inflammatory effects of berberine in *Mtb* infected BMDM. Berberine or vehicle-treated BMDM isolated from C57BL/6 mice were infected with *Mtb.* **(A)** IL-6, **(B)** TNF measured in the supernatants 2 days and 5 days post-infection and **(C)** accumulation of intracellular ROS was measured by CellROX Green reagent at 2 days post-infection. **(D)** Ser536 phosphorylated p65, total p65, Thr183/Tyr185 phosphorylated JNK, total JNK, and housekeeping control GAPDH levels were assayed by Western Blot in berberine or vehicle pre-treated BMDM at 30, 60 and 120 minutes post *Mtb* infection. **(E)** Human monocyte-derived macrophages from male and female donors (n = 3 each) were infected to determine mycobacterial growth at the indicated time points. Data represented as mean ± SEM of four replicates and representative of two experiments. Statistical significance was analysed by a two-tailed unpaired Student’s t-test defining differences in all groups as significant *P ≤ 0.05; **P ≤ 0.01.

We investigated whether berberine has an impact on the survival of *Mycobacterium tuberculosis* (*Mtb*) in liquid broth and macrophages. First, we assessed whether berberine has a direct anti-mycobacterial effect in a liquid culture medium supplemented with a range of concentrations for 12 days. We found that berberine had no mycobactericidal effect on the axenic growth of *Mtb* in a concentration range from 3.9-250 µM (**Figure 1A**). We then determine the cytotoxic effect of berberine on primary murine macrophages by Cell Titer Blue assay. BMDM were pre-treated with 2-fold concentrations of berberine from 0 to 2000 µM for 6 days. Macrophages treated with berberine at 62.5 µM and above displayed a significant cytotoxic effect. The highest concentration which had no cytotoxic effect was 31.25 µM and we used 30 µM used for subsequent intracellular assays (**Figure 1B**). Mouse BMDM were pre-treated with 30 µM overnight to determine the intracellular growth of *Mtb* in a time-kinetic manner. We found that berberine treatment had no effect on the uptake of Mtb (day 0) and showed a significant decrease in *Mtb* growth at 2 and 5 days post-infection (**Figure 1C**). The increased mycobacterial killing ability of macrophages was similar in macrophages treated with berberine after phagocytosis of *Mtb* (**Supplementary Figure 1A**). To further probe *Mtb* infected macrophage modulation upon berberine treatment, interestingly resulted in a higher percentage of MHCII and CD80 (**Figure 1D**) and higher expression of CD80 (**Supplementary Figure 1B**). Furthermore, berberine treatment resulted in decreased levels of nitric oxide (NO) (**Figure 1E**), pro-inflammatory cytokines IL-1α (**Figure 1F**), IL-6 (**Figure 2A**); while it did not effect TNFα (**Figure 2B**) levels *in vitro*. Berberine can exert its antibacterial effects through the increase of reactive oxygen species (ROS), although there are studies also reporting antioxidant activities of berberine (27–29). We have measured intracellular ROS levels of berberine-treated murine macrophages which showed no change in ROS accumulation (**Figure 2C**). In line with the previous reports, we observed decreased phosphorylation of p65 subunit of NF-κB and c-Jun N terminal kinase (JNK) (**Figure 2D**). Furthermore, we assessed the influence of berberine on the intracellular growth of *Mtb* in human monocyte-derived macrophages (MDM). Considering the sex differences in monocyte or MDM response (30), we measured the bacterial killing effect of berberine supplementation in female and male donor-derived MDM. Similar to mouse macrophages, both female and male MDM significantly decreased intracellular *Mtb* growth upon berberine treatment (**Figure 2E**). These results show that berberine treatment decreased *Mtb* growth in mouse and human macrophages possibly by modulating the antimicrobial capacity of the host; while paradoxically downregulating pro-inflammatory responses.

### Berberine as an Adjunctive Therapy Decreased the Lung Pathology During *Mtb* Infection in Mice

**Figure 3:**
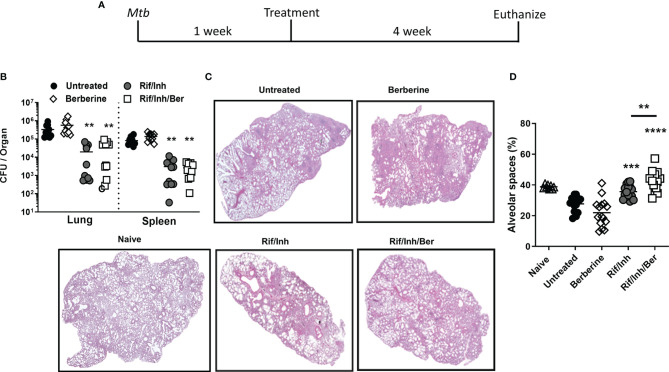
Berberine as an adjunctive therapy against *Mtb* infection in C57BL/6 mice. **(A)** Animals were infected with *Mtb* (100CFU) *via* aerosol inhalation. After 1 week of infection, mice were either left untreated or treated with berberine (1 mg/ml) or rifampin/isoniazid (0.1 mg/ml) and rifampin/isoniazid in combination with berberine in drinking water for 4 weeks as shown in the layout. **(B)** Mycobacterial burdens in the lung and spleen. **(C)** Representative lung section for pathology. **(D)** Quantification of free alveolar air spaces in the lungs at 5 weeks post-infection. Data is shown from pooled two experiments (n=10 per group) and the line denotes mean value, analysed by one-way ANOVA with Tukey *post-hoc* test defining differences in all groups as significant **P ≤ 0.01; ***P ≤ 0.001; ****P ≤ 0.0001. Asterisks without the line show the significance compared to the untreated group.

To investigate the biological relevance of our findings in macrophages, we determined whether berberine as an adjunctive therapy will have a host-protective effect *in vivo*. Oral administration of berberine is deemed safe since no mortality was observed in mice administered with as high as 20.8 g/kg of body weight due to extremely poor absorption in the gut, rapid and extensive metabolism (31, 32). Owing to low oral bioavailability, short-term and chronic berberine administration have not resulted in concerning adverse effects in the clinic (33). To evaluate the potential of berberine in TB therapy, mice were infected with a low dose (100CFU, *Mtb* H37Rv) by aerosol inhalation. One week after infection, mice were treated with berberine and rifampicin/isoniazid alone or in combination for 4 weeks *ad libitum* (**Figure 3A**). Berberine treatment groups consumed slightly less water; however, had no differences in body weight change (**Supplementary Figure 2A**). Moreover, serum alanine transaminase (ALT) and aspartate transaminase (AST) were similar between the groups after 4 weeks of treatment, indicating that berberine had no liver toxicity though there was a reduced trend of AST and ALT levels in the Rif/Inh/Ber group (**Supplementary Figure 2B**). After 4 weeks of treatment, mice were euthanized to determine mycobacterial burdens and lung pathology. We found that berberine alone or as an adjunctive to rifampicin/isoniazid had no effect on the lung and spleen mycobacterial burdens (**Figure 3B**). However, lung sections revealed that berberine used as an adjunct to first-line anti-TB drugs, INH and RIF, significantly decreased the lung tissue pathology (**Figure 3C**). Furthermore, we quantified the free alveolar spaces in these sections indeed revealed that berberine as an adjunct significantly increased the non-inflamed alveolar spaces in the lungs when compared to mice treated with INH/RIF alone or untreated mice (**Figure 3D**). These results demonstrate that berberine as an adjunct to first-line drugs decreased lung pathology during tuberculosis.

### Berberine as an Adjunctive Therapy Modulates Immune Cell Recruitment in the Lungs During *Mtb* Infection in Mice

**Figure 4:**
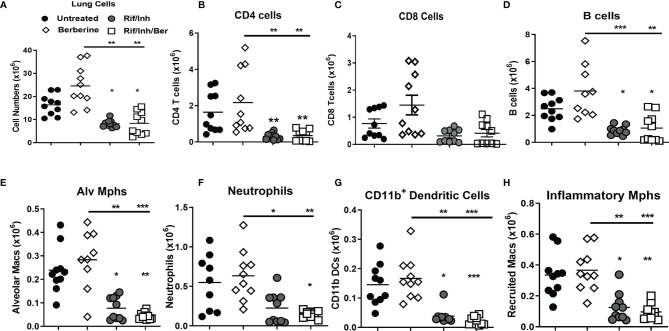
Effect of berberine on lung immune cells populations as an adjunctive therapy during *Mtb* infection in C57BL/6 mice. Single-cell suspension of lungs was analyzed for immune cell populations by flow cytometry at 5 weeks post-infection; **(A)** Total lung cells, **(B)** CD4 T cells (CD19^-^CD3^+^CD4^+^), **(C)** CD8 T cells (CD19^-^CD3^+^CD8^+^), **(D)** B cells (CD19^+^CD3^-^), **(E)** Alveolar macrophages (Siglec-F^+^CD11c^+^), **(F)** Neutrophils (Gr1^+^Siglec-F^-^Cd11c^-^), **(G)** Dendritic cells (CD11c^+^CD11b^+^MHCII^+^) and **(H)** inflammatory macrophages (CD11b^+^CD11c^-^MHCII^+^). Data is shown from pooled two experiments (n = 10 per group) and the line denotes mean value analysed by Kruskal-Wallis test with Dunn’s multiple comparisons correction defining differences in all groups as significant *P ≤ 0.05; **P ≤ 0.01; ***P ≤ 0.001. Asterisks without the line below show the significance compared to the untreated group.

We then assessed whether berberine as an adjunct treatment influences lung immune cell recruitment during *Mtb* infection. A single-cell suspension of lungs was prepared to analyse immune cell recruitment by flow cytometry. We found that mice treated with Rif/Inh or Rif/Inh/Ber showed decreased lung cell numbers (**Figure 4A**). The cell suspension was subjected to flow antibody staining using markers for phenotyping of lymphocytes and myeloid cell compartments. Berberine as an adjunct therapy decreased CD4^+^ and CD8^+^ T cells (**Figures 4B, C**) to the same level as Rif/Inh group. Similar to T cells, B cells (**Figure 4D**) were also decreased in mice treated with Rif/Inh/Ber to a similar extent of Rif/Inh group. Amongst myeloid cells, berberine as an adjunct decreased alveolar macrophages when compared to untreated, similar to rifampicin/isoniazid treated mice (**Figure 4E**). Neutrophils are a major contributor to lung pathology in tuberculosis (34, 35). Mice treated with rifampicin/isoniazid decreased neutrophils in the lungs (**Figure 4F**), most probably due to reduced lung burdens. Berberine combined with rifampicin/isoniazid further significantly decreased the neutrophils when compared to mice treated with berberine alone (**Figure 4F**). This may explain the observed reduced lung pathology in this group of mice. Furthermore, mice treated with rifampicin/isoniazid alone and antibiotics with berberine displayed reduced conventional CD11b^+^ dendritic cells (**Figure 4G**) and inflammatory macrophages (**Figure 4H**) when compared to untreated or berberine alone animals. These results suggested that berberine as an adjunct to antibiotic therapy decreased lymphoid and myeloid cell recruitment in the lungs during tuberculosis.

### Berberine as an Adjunctive Therapy Decreases Certain Inflammatory Cytokines in the Lungs During *Mtb* Infection in Mice

**Figure 5:**
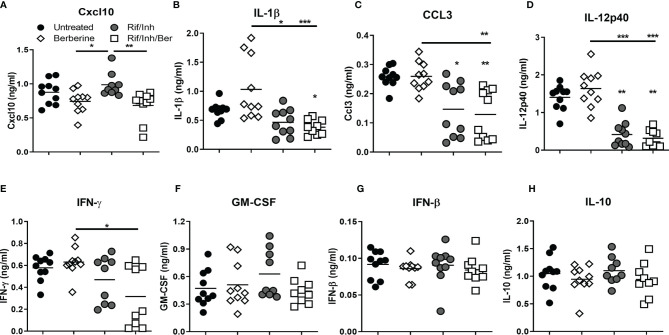
Effect of berberine on lung cytokines as an adjunctive therapy during *Mtb* infection in C57BL/6 mice. Lung homogenates were analyzed for the cytokines and chemokine by ELISA at 5 weeks post-infection; **(A)** CXCL10, **(B)** IL-1β, **(C)** CCL3, **(D)** IL-12p40, **(E)** IFNγ, **(F)** GM-CSF, **(G)** IFN-β and **(H)** IL-10. Data is shown from pooled two experiments (n = 10 per group) and the line denotes mean value, analysed by Kruskal-Wallis test with Dunn’s multiple comparisons correction defining differences in all groups as significant *P ≤ 0.05; **P ≤ 0.01; ***P ≤ 0.001. Asterisks without the line below show the significance compared to the untreated group.

We next assessed the cytokine levels in lung homogenates by ELISA at 4 weeks post-treatment. The analysis revealed that mice treated with berberine as an adjunct significantly reduced CXCL-10 (**Figure 5A**), while there was a decreasing trend towards IL-1β (**Figure 5B**) and CCL3 levels (**Figure 5C**) when compared to antibiotics alone. CXCL10 is an inflammatory chemokine that activates cells to increase inflammatory lung damage and is highly expressed in patients with active TB (36). Rif/Inh treatment decreased IL-12p40 levels drastically; however adjunct berberine treatment did not promote the reduction of IL-12p40 levels (**Figure 5D**). Moreover, adjunct berberine treatment did not affect IFNγ, GM-CSF, IFN-β and IL-10 levels in the lung (**Figures 5E–H**). Interestingly, CCL3 and IFNγ levels showed a bimodal distribution similar to lung CFU (**Figure 3B**), indicating that lung bacterial burdens are the major driver in the production of these cytokines/chemokines. Together, these results suggest that berberine as an adjunct decreases certain chemokine/cytokine production in the lungs during *Mtb* infection.

### Onset of Late Berberine Treatment Does Not Effect Its Adjuvant Potential While Exerting Stronger Responses in the Spleen

**Figure 6:**
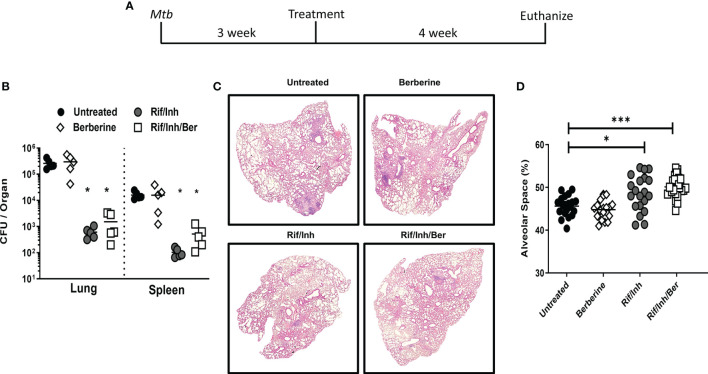
Late-onset of berberine adjunctive therapy against *Mtb* infection in C57BL/6 mice. **(A)** Animals were infected with *Mtb* (100CFU) *via* aerosol inhalation. After 3 weeks of infection, mice were either left untreated or treated with berberine (1 mg/ml) or rifampin/isoniazid (0.1 mg/ml) and rifampin/isoniazid in combination with berberine in drinking water for 4 weeks as shown in the layout. **(B)** Mycobacterial burdens in the lung and spleen. **(C)** Representative lung section for pathology. **(D)** Quantification of free alveolar air spaces in the lungs at 4 weeks post-treatment. Data is shown representative of two experiments (n = 5 per group) and the line denotes mean value, analysed by Brown-Forsythe and Welch ANOVA test with Tamhane T2 *post-hoc* test defining differences in all groups as significant *P ≤ 0.05; ***P ≤ 0.001. Asterisks without the line below show the significance compared to the untreated group.

**Figure 7:**
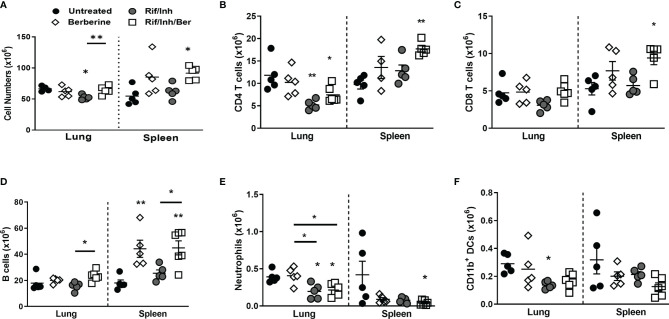
Effect of late-onset berberine adjunctive therapy on lung and spleen immune cell populations during *Mtb* infection in C57BL/6 mice. Single-cell suspension of lungs and spleens were analyzed for immune cell populations by flow cytometry at 4 weeks post-treatment; **(A)** Total lung and spleen cells, **(B)** CD4 T cells (CD19^-^CD3^+^CD4^+^CD8^-^), **(C)** CD8 T cells (CD19^-^CD3^+^CD8^+^CD4^-^), **(D)** B cells (CD19^+^CD3^-^), **(E)** Neutrophils (CD11b^+^Ly6G^+^), **(F)** CD11b Dendritic cells counts (CD11c^+^CD11b^+^MHCII^+^). Data is shown representative of two experiments (n = 5 per group) and the line denotes mean value, analysed by one-way ANOVA with Tukey *post-hoc* test **(A–D)** and Kruskal-Wallis test with Dunn’s multiple comparisons correction **(E, F)** defining differences in all groups as significant *P ≤ 0.05; **P ≤ 0.01. Asterisks without the line below show the significance compared to the untreated group.

H37Rv *Mtb* strain grows exponentially during the first three-four weeks of infection in the lungs of C57BL/6 mice and bacterial burden stabilizes after this stage which coincides with the arrival of antigen-specific T cells in the lungs (37, 38). We, therefore, were prompted to adapt adjunct berberine treatment at peak bacterial burdens levels in the lung i.e. 3 weeks after infection (**Figure 6A**). Berberine alone treatment or adjunct berberine treatment did not affect lung and spleen bacterial burdens (**Figure 6B**); however, the adjunctive treatment resulted in decreased tissue involvement (**Figures 6C, D**) similar to berberine treatment started at 1 week after infection. However, adjunctive treatment resulted in increased lung and spleen total cell numbers (**Figure 7A**) which are driven mainly by increased CD4 T cells (**Figure 7B**) and B cell numbers (**Figure 7C**). There were also expanded CD4 T cell and CD8 T cell and B cell numbers in the spleens of the adjunctive treatment group and a similar trend was observed in the berberine alone treatment group (**Figures 7B, D**). Similar to earlier adjunct treatment, we observed decreased neutrophil numbers in the spleens and lungs of the adjunctive group (**Figure 7E**); however, conventional CD11b^+^ dendritic cells (**Figure 7F**) remain unaffected. To recapitulate *in vitro* macrophage activation results, we investigated MHCII expression levels in lung and spleen myeloid subsets. In the lungs, lower percentages of recruited interstitial macrophages in the adjunct group were MHCII positive, but CD169^+^ macrophages, red pulp macrophages and monocytes of berberine alone and adjunctive treatment groups consistently showed increased MHCII expression (**Supplementary Figure 2C**). It is shown that berberine influences caspase 3 activation and downstream apoptosis events in a cell-specific manner (39); we sought out *in vivo* activated caspase-3 levels in lung and spleen cells. The adjunctive treatment group showed decreased caspase-3 positive cells in the spleen, but this effect was not observed in the lungs (**Supplementary Figure 2D**). CXCR3 is the receptor for CXCL-10 and regulates the migration of antigen-specific Th1 cells into the lung (40). CXCR3^+^ CD4 T cells are efficient in localizing lung parenchyma around the lymphocytic cuff of TB granulomas and rarely in the myeloid core (41). We found that lung CD4 T cells in the antibiotic-treated groups have increased frequencies of CXCR3^+^ subsets and the adjunctive berberine treatment decreased the frequency of CXCR3^+^ CD4 T cells in the spleen compared to Rif/Inh group (**Supplementary Figures 3A, E**). Late onset of berberine adjunctive treatment did not change lung alveolar macrophage, recruited interstitial macrophage, lung monocyte-derived DC, spleen CD169^+^ macrophage, spleen red pulp macrophage or spleen CD8α^+^ DC cell numbers (**Supplementary Figures 3B–D, F–H**). In terms of lung and spleen cytokine/chemokine levels; lung IFNγ, lung and spleen CXCL10, lung GM-CSF, lung and spleen IFNβ levels remain unchanged (**Supplementary Figures 4A, D–F**). Antibiotic treatment groups showed reduced spleen IFNγ, spleen IL-1β and lung IL-12p40 levels (**Supplementary Figures 4A–C**). Interestingly, the berberine alone group increased lung and spleen IL-1β levels (**Supplementary Figure 4B**); a similar trend was also observed in the early treatment onset of berberine (**Figure 5B**). Aside from CXCL10 signaling, we also investigated whether IFNγ and GM-CSF signaling was affected by gene expression levels of IFNγ (Ifngr1) and GM-CSF receptors (Csf2ra) in total lung tissue by qPCR. Berberine alone suppressed Ifngr1 expression levels while Csf2ra expression did not change among different groups (**Supplementary Figure 4G**). Our data suggest that late-onset of berberine treatment still reduced neutrophil recruitment and beneficial against lung pathological responses, while spleen tissue was more affected by adjunctive berberine treatment.

### Berberine Decreases Bacterial Load and Pathology During *Mtb* Infection in C3HeB/FeJ Mice

We further investigated the host protective role of berberine in a more relevant model of tuberculosis. We used C3HeB/FeJ (Kramnik) mice, which recapitulates the progressive lung pathology of patients with TB. Mice were infected with *Mtb* Erdman *via* aerosol inhalation to deposit 100CFU in the lungs. After 3 weeks of infection, cohorts of mice were treated with either berberine or antibiotics or berberine combined with antibiotics (**Supplementary Figure 5A**). Four weeks following treatment, mice were euthanized to analyze the lung mycobacterial burden as CFU, the extent of inflammation as measured by immune cell recruitment and histopathological lung damage. The bacterial burden was similar between antibiotics and berberine as an adjunctive (**Supplementary Figure 5B**). Interestingly, berberine alone also significantly decreased lung mycobacterial burdens in Kramnik mice as opposed to C57BL/6 mice (**Supplementary Figure 5B**). Furthermore, H&E staining revealed that lung pathology is decreased in cohorts treated with berberine alone or in combination with antibiotics (**Supplementary Figure 5C**). Lung alveolar space quantification revealed that berberine alone did not effect lung pathology to a significant extent, however, berberine as adjunctive therapy increased the lung alveolar free space when compared to berberine alone or untreated group (**Supplementary Figure 5D**). We then assess the recruitment of immune cells in the lungs of these mice. In line with late onset in C57BL/6 mice, berberine as an adjunct significantly increased total cells harvest when compared to mice treated with antibiotics alone (**Supplementary Figure 5E**). Similarly, T cells (**Supplementary Figure 5F**), polymorphonuclear cells (**Supplementary Figure 5G**) and recruited monocytes (**Supplementary Figure 5H**) were significantly increased in mice treated with berberine as an adjunct when compared to animals treated with antibiotics alone. This may explain increased total lung cells harvested. Berberine, alone had no effect on neutrophil influx to the lung suggesting that this compound modulates inflammation without perturbing these innate cell recruitment. In contrast to C57BL/6 model, berberine treatment combined with antibiotics significantly increased neutrophil recruitment to the lung when compared to animals treated with antibiotics alone (**Supplementary Figure 5G**). Collectively, these results indicate that berberine as adjunctive therapy may offer enhanced protection against tuberculosis infection in an experimental model of progressive pulmonary TB.

## Discussion

Our findings demonstrate that berberine as an adjunct to rifampicin/isoniazid against tuberculosis decreased lung pathology in an experimental murine model of TB. The pleiotropic actions of berberine are mainly associated with its immunomodulatory properties through inhibition of NF-κB, MAPK and inhibition of pro-inflammatory cytokine production (16). We have also observed similar effects of berberine in *Mtb*-infected mouse macrophages *in vitro*. Intriguingly, berberine treatment pre- or post-*Mtb* infection increased the bactericidal activity of macrophages despite they released decreased levels of nitric oxide, IL-6, and IL-1α. Similar to murine macrophages, human macrophages also reduced mycobacterial growth following berberine treatment. The limitation is macrophages were not generated from CD16-negative selected monocytes which may result in mixed M1 and M2 macrophages, however, markers of classical and alternative activation are not very clear in human macrophages. Increased ROS levels contribute to the bacterial killing, we confirmed that decreased bacterial growth in macrophages was independent of ROS. In contrast, berberine had no direct inhibitory effect on *Mtb* but modulates host responses to mediate antimycobacterial effects. Our findings are in line with the report showed enhanced macrophage antibacterial activity even though berberine inhibits mRNA expression of iNOS, COX‐2, IL-1β, TNF and IL‐6 in LPS‐stimulated inner medullary collecting duct-3 cells by reducing NF‐kB activity (42). In atherosclerosis, berberine treatment inhibits inflammation in mouse macrophages (J774A.1) by inducing autophagy through AMPK/mTOR pathway (43) and uncoupling protein 2 in mice (44). Moreover, berberine inhibits the formation of foamy macrophages by enhancing LXRalpha‐ABCA1‐dependent cholesterol efflux in macrophages (45). Additionally, a berberine derivative is shown to induce lysosomal acidification through activation of transcription factor EB in methicillin-resistant *Staphylococcus aureus* and enteroinvasive *Escherichia coli* infected BMDM (46). Therefore, it is feasible that berberine treatment decreases the intracellular survival of *Mtb* in macrophages. Activation of macrophages by increased CD80 expression and increased frequencies of MHCII^+^ BMDM or splenic macrophages *in vivo* can be context-specific to *Mtb* infection. It has been previously reported that berberine pre-treatment did not change cell surface expression of CD80 and MHCII in naïve macrophages (47) or decreased CD80 expression in lipopolysaccharide (LPS) stimulated bone marrow-derived dendritic cells (18). Increased macrophage activation and macrophage *Mtb* killing mechanisms, while decreased proinflammatory gene expression and NF-κB and JNK kinase activation shows that berberine has pleiotropic and context-specific functions in macrophages.

Augmenting macrophage activation and bacterial killing mechanisms while reducing inflammation could increase the immunomodulatory potential of berberine during TB disease. Moreover, berberine immunotherapy as an adjunct had a beneficial effect for the host by decreasing lung pathology in the treated C57BL/6 after 4 weeks of therapy. However, berberine alone or in combination did not affect lung mycobacterial burdens. Within 2 weeks *Mtb* disseminates from the lungs to other organs for example spleen and liver (48). Considering this berberine may target dissemination to other organs during treatment. Similar to lungs, berberine had no effect on mycobacterial burdens in the spleen. Interestingly, we found that berberine showed stronger effects on macrophage activation and B lymphocyte numbers in the spleen. Increased B cell numbers in the spleen were also reported in the mouse leukemia model before (49). It will be interesting to study whether berberine affects B-cell development in the bone marrow or reduces B-cell turnover. Overall, this study revealed that berberine treatment reduced deleterious lung pathological consequences during TB.

Berberine also has anti-parasitic (50), -viral (51), -fungal (52) and -helminth activity (53). Previously, administration of berberine with isoniazid protects against liver injury caused by oxidative stress and inflammation in rats by suppressing NF‐kB, iNOS, the pro‐inflammatory cytokines and upregulating PPAR-γ (54). We found that berberine has an adjunctive effect on rifampicin/isoniazid-induced control of lung inflammation in *Mtb*-infected mice. The synergistic effects of berberine have also been reported with commonly used antibiotics against Methicillin-Resistant *Streptococcus aureus* (MRSA) (55) and fluconazole‐resistant *Candida albicans* (56). Berberine has been proven for its antibacterial activity against a broad spectrum of microbial pathogens (57) and exhibited sub-MICs on conventional antimicrobial agents such as ampicillin, azithromycin, cefazolin, and levofloxacin (58). The primary antibacterial mechanism of berberine is due to inhibition of the cell division protein FtsZ (59). Berberine has a synergistic effect with some common antibiotics especially with linezolid, cefoxitin, and erythromycin (60). The direct microbicidal effect of berberine appears to inhibit biofilm formation (61) and synthesis of DNA (57). In our study, we could not detect ROS levels *in vivo* due to the short half-life of ROS; however, *in vitro* BMDM showed that berberine does not show antioxidant properties during *Mtb* infection. Berberine increases the antibacterial properties of macrophages and we observed a similar trend in the susceptible Kramnik mouse model when the bacterial burdens were high in the lung. We ruled out the possibility of hepatotoxic effects of berberine by probing serum ALT and AST levels. The stronger effects of berberine were seen in the lung pathology in C57BL/6 mice. The decreased lung pathology was associated with fewer neutrophil recruitment both in the lungs and spleen. Additionally, berberine showed immunomodulatory properties *in vivo* by increased macrophage activation and B cell numbers. Overall, the data suggest that the effects of adjunctive berberine treatment are pleiotropic during *Mtb* infection.

We then further validated our findings in a susceptible and highly inflammatory Kramnik (C3HeB/FeJ) mice, a highly relevant model, which recapitulates human lung lesions and a comprehensive model to study tuberculosis immunopathology (62). Remarkably in these animals, berberine alone was able to significantly reduce lung mycobacterial burden, which might be attributed to higher susceptibility of this animal model in comparison to C57BL/6 mice. However, no adjunct effect on bacterial load was observed in mice treated with a combination of berberine and antibiotics; the latter was consistent with the C57BL/6 model. Moreover, rifampicin/isoniazid alone significantly increased lung alveolar air spaces compared to mice treated with berberine alone. However, mice treated with rifampicin/isoniazid in combination with berberine did not further increase lung alveolar spaces when compared to antibiotic only group. Furthermore, mice treated with rifampicin/isoniazid in combination with berberine showed increased lung cell numbers, T cells, polymorphonuclear cells and recruited monocytes in the lungs. These findings mirrored our observations with C57BL/6 mice when treated 3 weeks post *Mtb* infection except for the effect on neutrophils. This indicated that berberine had an adjunct but not additive or synergistic effect on lung inflammation in C57BL/6 mice but not susceptible Kramnik mice.

The absence of the adjunctive effect of berberine in Kramnik mice points out that preventing neutrophilic inflammation is the main driver of anti-inflammatory effects of berberine in C57BL/6 mice. Previously, forward genetics analyses revealed that Kramnik mice have a susceptible allele of super susceptibility to tuberculosis (sst1) locus on mouse chromosome 1, unlike C57BL/6 counterparts which harbor the resistant allele (63) Sp140 gene was recently identified to confer resistance to C57BL/6 mouse and it is found to negatively regulate Type I IFN responses (64). Type I IFNs are shown to drive pathological inflammation in tuberculosis especially in Kramnik mice through neutrophilia (35, 65). In our experiments, we have seen that the early or late onset of adjunctive berberine treatment did not effect IFNβ levels in C57BL/6 mice, hence Type-1 IFN dominated neutrophilia was probably not inhibited in Kramnik mice. Targeting Type I IFN network by neutralizing antibodies with berberine adjunctive therapy would be a future study to understand the extent of berberine immunomodulation and anti-inflammatory effects in adjuvant therapy in Kramnik mice.

In summary, we report here that berberine as an adjunctive therapy decreased lung inflammation by targeting immune cell recruitment and reducing inflammatory cytokines. The lack of the development of granulomatous lung pathology with caseous necrosis resembling the human TB disease in the C57BL/6 model and Kramnik experiment further warrants studies starting treatment in much later time points in Kramnik mice or nonhuman primates (NHP) before testing in patients. Another important drawback of berberine adjunctive therapy can be attributed to its low absorption rates in the gut (66). Therefore, future studies with hyperinflammatory clinical Mtb strains in Kramnik mouse and NHP models are warranted to consider berberine or its derivatives with increased oral bioavailability as a potential adjunct therapy to current first-line anti-TB drugs in the clinic.

## Data Availability Statement

The original contributions presented in the study are included in the article/**Supplementary Material**. Further inquiries can be directed to the corresponding author.

## Ethics Statement

The animal study was reviewed and approved by Animal Ethics Committee (Permit Number: 015/040), Faculty of Health Sciences, University of Cape Town, Cape Town, South Africa.

## Author Contributions

MO, JC, RH, MS, RM, RG, BM, and SP: designing research studies, conducting experiments, acquiring data, analyzing data. BM, HS, and FB: resources and funding for the research. SP and MO writing the manuscript. All authors contributed to the article and approved the submitted version.

## Funding

This work was supported by the Global Health Innovative Technology (GHIT) Fund, Japan post-doctoral fellowship to SP. ICGEB Arturo Falaschi post-doctoral and EDCTP post-doctoral fellowship(s) to MO. South African Medical Research Council (SAMRC) Unit on Immunology of Infectious Diseases, National Research Funding (NRF) South Africa and the South African Research Chair Initiative (SARChi) to FB. GHIT research grant awarded to HS and FB. The research was conducted in the BSL3 platform supported by core funding from the Wellcome Trust (203135/Z/16/Z).

## Conflict of Interest

The authors declare that the research was conducted in the absence of any commercial or financial relationships that could be construed as a potential conflict of interest.

## Publisher’s Note

All claims expressed in this article are solely those of the authors and do not necessarily represent those of their affiliated organizations, or those of the publisher, the editors and the reviewers. Any product that may be evaluated in this article, or claim that may be made by its manufacturer, is not guaranteed or endorsed by the publisher.

## Supplementary Material

Supplementary Figure 1 Bactericidal and immunomodulatory effects of berberine on *Mtb* infected macrophages. **(A)** BMDM from C57BL/6 mice were either pre-treated with berberine (30 µM) overnight or treatment started 4 hours post-infection. Cells were infected with *Mtb* (MOI = 1) to determine the growth in 2 days and 5 days post-infection (dpi); 4 hours post-infection CFU was assayed to measure the differences in bacterial uptake. **(B)** MHCII and CD80 mean fluorescence intensity (MFI) was measured in vehicle or berberine treated *Mtb* infected BMDM to check protein abundance on the surface. Data represented as mean ± SEM of four replicates and representative of two experiments, analysed by a two-tailed unpaired Student’s t-test defining differences in all groups as significant *P ≤ 0.05; **P ≤ 0.01, ***P ≤ 0.001.

Supplementary Figure 2 Immunomodulatory effects of berberine as an adjunctive therapy during *Mtb* infection in C57BL/6 mice **(A)** Water consumption was measured after every water change to estimate drug uptake per mouse per day. **(B)** Serum ALT and AST levels were measured 4 weeks post TB treatment to infer liver cytotoxicity and hepatoprotective effects of berberine. **(C)** MHCII expressing lung alveolar macrophages, interstitial macrophages and monocyte-derived DCs; spleen CD169^+^ macrophages, red pulp macrophages and monocytes after 4 weeks treatment in late-onset of berberine adjunctive therapy. The data is presented as the percentage of the parent population. **(D)** Activated caspase 3 flow cytometry as a marker of apoptosis. Data is shown as Caspase-3^+^ total live cells in the lungs and spleen of mice 4 weeks post TB treatment in the late-onset adjunctive berberine treatment. Data is shown representative of two experiments (n = 5 per group) and the line denotes mean value, analysed by Kruskal-Wallis test with Dunn’s multiple comparisons correction **(A, B)** and one-way ANOVA with Tukey *post-hoc* test **(C, D)** defining differences in all groups as significant *P ≤ 0.05; **P ≤ 0.01. Asterisks without the line below show the significance compared to the untreated group.

Supplementary Figure 3 Effect of late-onset berberine adjunctive therapy on lung and spleen immune cells populations during *Mtb* infection in C57BL/6 mice. **(A)** Lung CXCR3^+^ CD4 and CD8 T cell frequencies, **(B)** lung alveolar macrophage (SiglecF^+^CD11c^+^CD64^+^), **(C)** lung interstitial macrophages (CD64^+^CD11b^+^CD11c^-^SiglecF^-^), **(D)** lung monocyte-derived DC counts (CD64^+^CD11b^+^CD11c^+^), **(E)** spleen CXCR3^+^ CD4 and CD8 T cell frequencies, **(F)** spleen CD169^+^ macrophage (CD11b^+^CD169^+^CD11c^-^) **(G)** spleen red pulp macrophage (F4/80^+^CD11b^low^CD169^-^CD11c^-^), **(H)** spleen CD8 DC counts (CD11c^+^MHCII^+^CD8^+^CD11b^-^). Data is shown representative of two experiments (n = 5 per group) and the line denotes mean value, analysed by one-way ANOVA with Tukey *post-hoc* test **(A–C, E–F)** and Kruskal-Wallis test with Dunn’s multiple comparisons correction (D, G) defining differences in all groups as significant *P ≤ 0.05; **P ≤ 0.01; ***P ≤ 0.001. Asterisks without the line below show the significance compared to the untreated group.

Supplementary Figure 4 Effect of late-onset of berberine adjunctive therapy on lung and spleen cytokines/chemokines and receptor gene expression during *Mtb* infection in C57BL/6 mice. Lung and spleen homogenates were analyzed for the cytokines and chemokines by ELISA at 4 weeks post-treatment; **(A)** IFNγ, **(B)** IL-1β, **(C)** IL-12p40, **(D)** CXCL10, **(E)** GM-CSF, and **(F)** IFN-β. **(G)** Total RNA was extracted from lung tissue and qPCR was performed to measure Ifngr1 and Csf2ra levels. Hprt housekeeping gene was used for normalization and fold change differences were calculated by using untreated as the reference sample. Data is shown representative of two experiments (n = 5 per group) and the line denotes mean value, analysed by one-way ANOVA with Tukey *post-hoc* test **(A–F)** and Kruskal-Wallis test with Dunn’s multiple comparisons correction **(G)** defining differences in all groups as significant *P ≤ 0.05; **P ≤ 0.01; ***P ≤ 0.001. Asterisks without the line below show the significance compared to the untreated group.

Supplementary Figure 5 Berberine as an adjunctive therapy against *Mtb* infection in C3HeB/FeJ (Kramnik) mice. **(A)** Mice were infected with *Mtb* Erdman (100 CFU) *via* aerosol inhalation. After 3 weeks of infection, mice were either left untreated or treated with berberine (1 mg/ml) or rifampin/isoniazid (0.1 mg/ml) and rifampin/isoniazid with berberine in drinking water for 4 weeks as shown in the layout. **(B)** Mycobacterial burdens in the lungs. **(C)** Representative lung section for pathology evaluated by H&E staining. **(D)** Quantification of free alveolar air spaces. Single-cell suspension of the lung was analyzed for immune cell populations by flow cytometry. **(E)** Total lung cells, **(F)** T cells (CD3^+^CD11b^-^Ly6G^-^Ly6C^-^), **(G)** polymorphonuclear cells (CD11b^+^Ly6G^+^Ly6C^+^) and **(H)** inflammatory monocytes (CD11b^+^Ly6G^-^Ly6C^+^) in the lungs at 4 weeks post-treatment. Data represented as mean ± SEM of n = 3-4 mice per group from one experiment, analysed by one-way ANOVA with Tukey *post-hoc* test defining differences in all groups as significant *P ≤ 0.05; **P ≤ 0.01; ***P ≤ 0.001.
