# Supplementary figures and images for: Evaluation of Berberine as an Adjunct to TB Treatment
Source: Front Immunol. 2021 Oct 20;12:656419. doi: 10.3389/fimmu.2021.656419 (PMC8563784; doi:10.3389/fimmu.2021.656419)

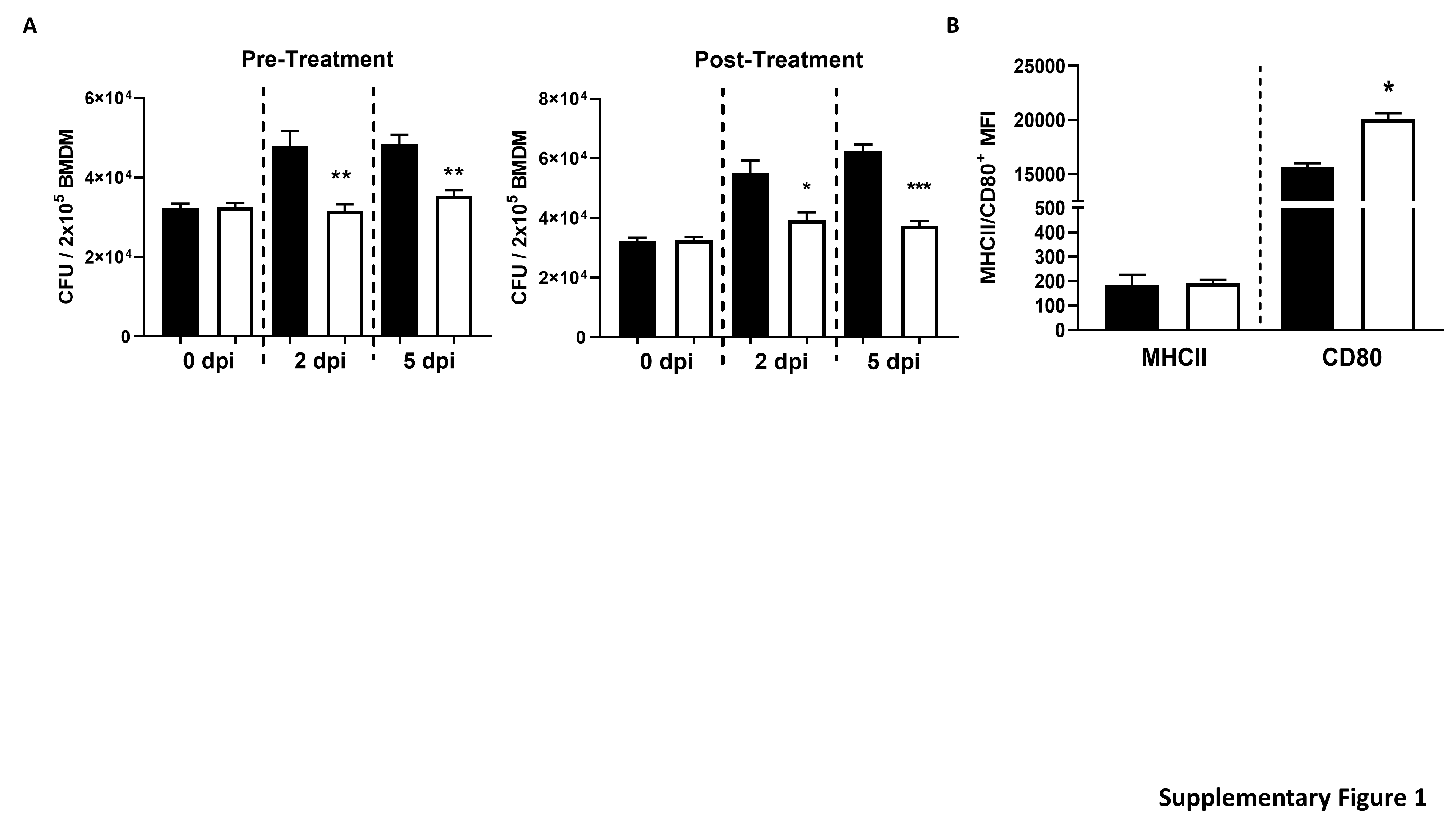

Supplement: Supplementary Figure 1 — Bactericidal and immunomodulatory effects of berberine on Mtb infected macrophages. (A) BMDM from C57BL/6 mice were either pre-treated with berberine (30 µM) overnight or treatment started 4 hours post-infection. Cells were infected with Mtb (MOI = 1) to determine the growth in 2 days and 5 days post-infection (dpi); 4 hours post-infection CFU was assayed to measure the differences in bacterial uptake. (B) MHCII and CD80 mean fluorescence intensity (MFI) was measured in vehicle or berberine treated Mtb infected BMDM to check protein abundance on the surface. Data represented as mean ± SEM of four replicates and representative of two experiments, analysed by a two-tailed unpaired Student’s t-test defining differences in all groups as significant *P ≤ 0.05; **P ≤ 0.01, ***P ≤ 0.001. [file Image_1.tif]

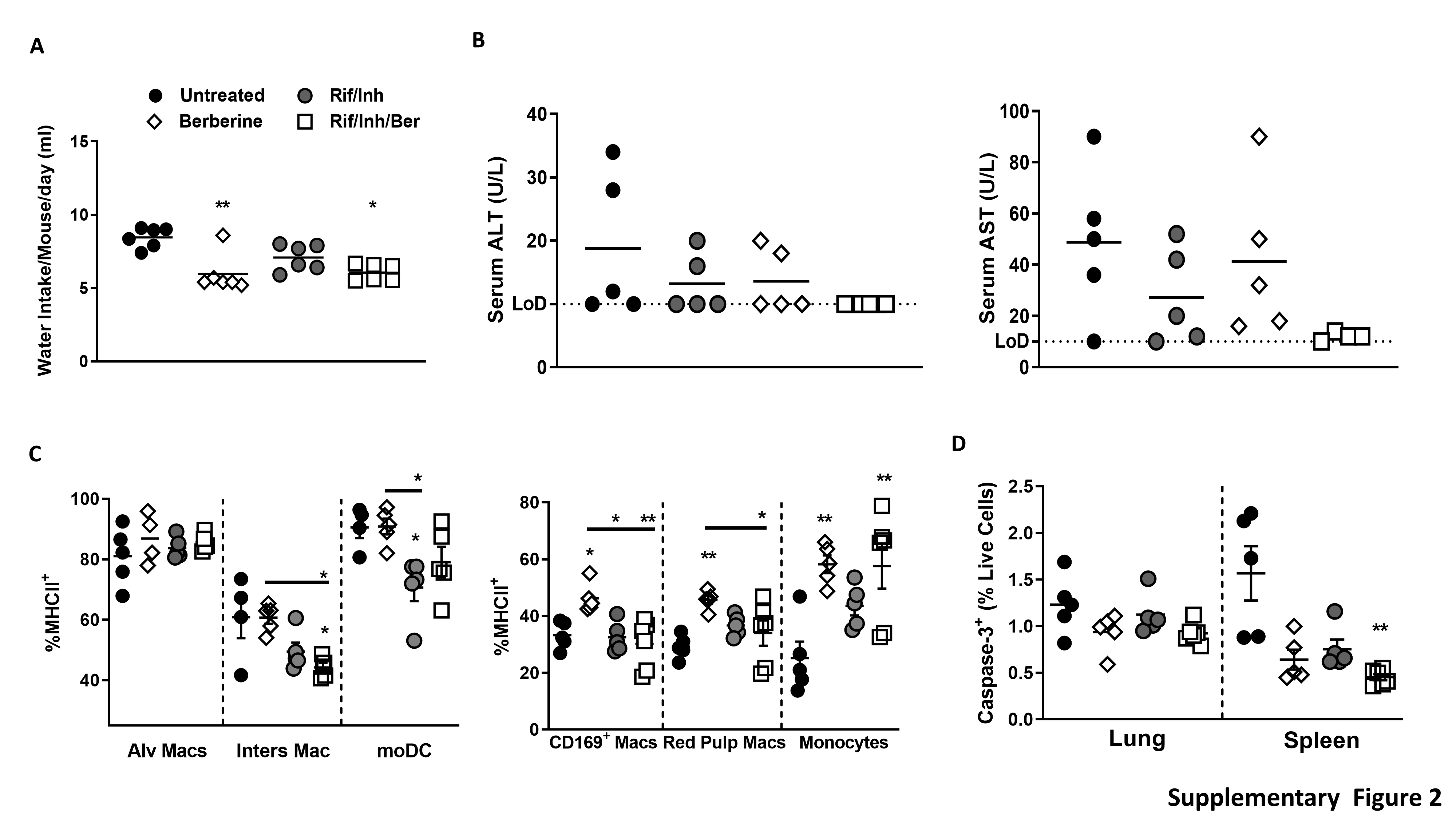

Supplement: Supplementary Figure 2 — Immunomodulatory effects of berberine as an adjunctive therapy during Mtb infection in C57BL/6 mice (A) Water consumption was measured after every water change to estimate drug uptake per mouse per day. (B) Serum ALT and AST levels were measured 4 weeks post TB treatment to infer liver cytotoxicity and hepatoprotective effects of berberine. (C) MHCII expressing lung alveolar macrophages, interstitial macrophages and monocyte-derived DCs; spleen CD169+ macrophages, red pulp macrophages and monocytes after 4 weeks treatment in late-onset of berberine adjunctive therapy. The data is presented as the percentage of the parent population. (D) Activated caspase 3 flow cytometry as a marker of apoptosis. Data is shown as Caspase-3+ total live cells in the lungs and spleen of mice 4 weeks post TB treatment in the late-onset adjunctive berberine treatment. Data is shown representative of two experiments (n = 5 per group) and the line denotes mean value, analysed by Kruskal-Wallis test with Dunn’s multiple comparisons correction (A, B) and one-way ANOVA with Tukey post-hoc test (C, D) defining differences in all groups as significant *P ≤ 0.05; **P ≤ 0.01. Asterisks without the line below show the significance compared to the untreated group. [file Image_2.tif]

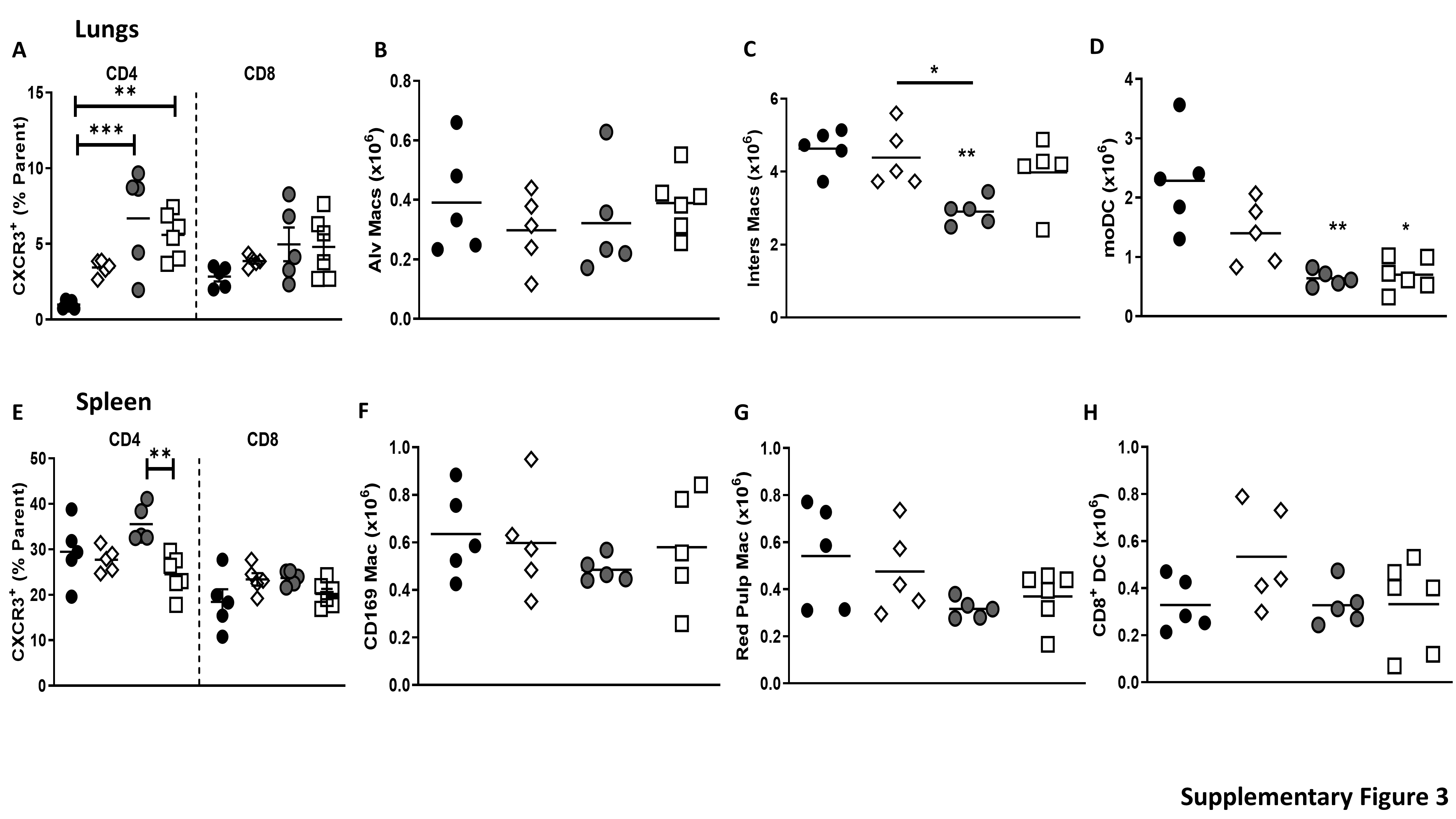

Supplement: Supplementary Figure 3 — Effect of late-onset berberine adjunctive therapy on lung and spleen immune cells populations during Mtb infection in C57BL/6 mice. (A) Lung CXCR3+ CD4 and CD8 T cell frequencies, (B) lung alveolar macrophage (SiglecF+CD11c+CD64+), (C) lung interstitial macrophages (CD64+CD11b+CD11c-SiglecF-), (D) lung monocyte-derived DC counts (CD64+CD11b+CD11c+), (E) spleen CXCR3+ CD4 and CD8 T cell frequencies, (F) spleen CD169+ macrophage (CD11b+CD169+CD11c-) (G) spleen red pulp macrophage (F4/80+CD11blowCD169-CD11c-), (H) spleen CD8 DC counts (CD11c+MHCII+CD8+CD11b-). Data is shown representative of two experiments (n = 5 per group) and the line denotes mean value, analysed by one-way ANOVA with Tukey post-hoc test (A–C, E–F) and Kruskal-Wallis test with Dunn’s multiple comparisons correction (D, G) defining differences in all groups as significant *P ≤ 0.05; **P ≤ 0.01; ***P ≤ 0.001. Asterisks without the line below show the significance compared to the untreated group. [file Image_3.tif]

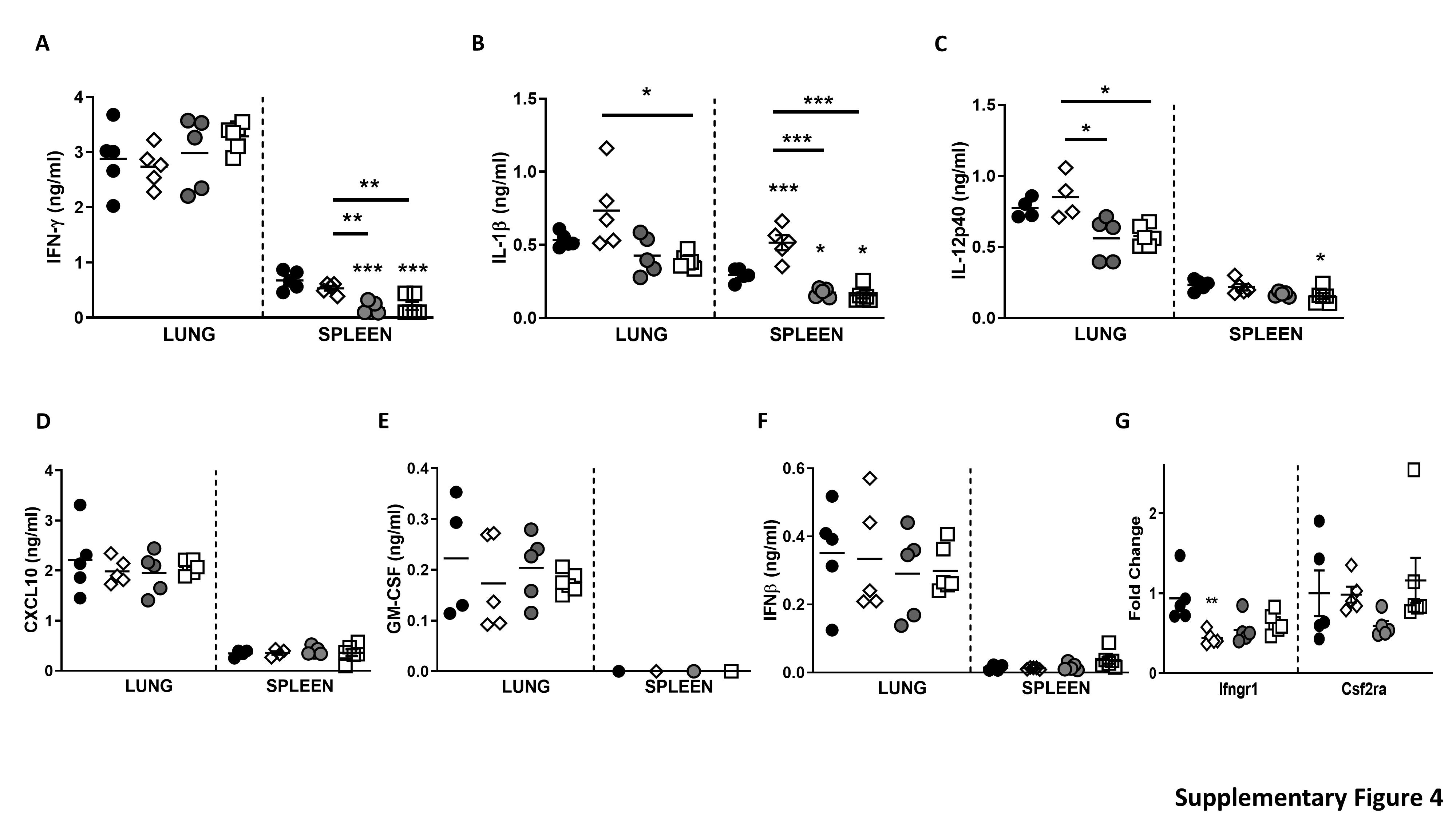

Supplement: Supplementary Figure 4 — Effect of late-onset of berberine adjunctive therapy on lung and spleen cytokines/chemokines and receptor gene expression during Mtb infection in C57BL/6 mice. Lung and spleen homogenates were analyzed for the cytokines and chemokines by ELISA at 4 weeks post-treatment; (A) IFNγ, (B) IL-1β, (C) IL-12p40, (D) CXCL10, (E) GM-CSF, and (F) IFN-β. (G) Total RNA was extracted from lung tissue and qPCR was performed to measure Ifngr1 and Csf2ra levels. Hprt housekeeping gene was used for normalization and fold change differences were calculated by using untreated as the reference sample. Data is shown representative of two experiments (n = 5 per group) and the line denotes mean value, analysed by one-way ANOVA with Tukey post-hoc test (A–F) and Kruskal-Wallis test with Dunn’s multiple comparisons correction (G) defining differences in all groups as significant *P ≤ 0.05; **P ≤ 0.01; ***P ≤ 0.001. Asterisks without the line below show the significance compared to the untreated group. [file Image_4.tif]

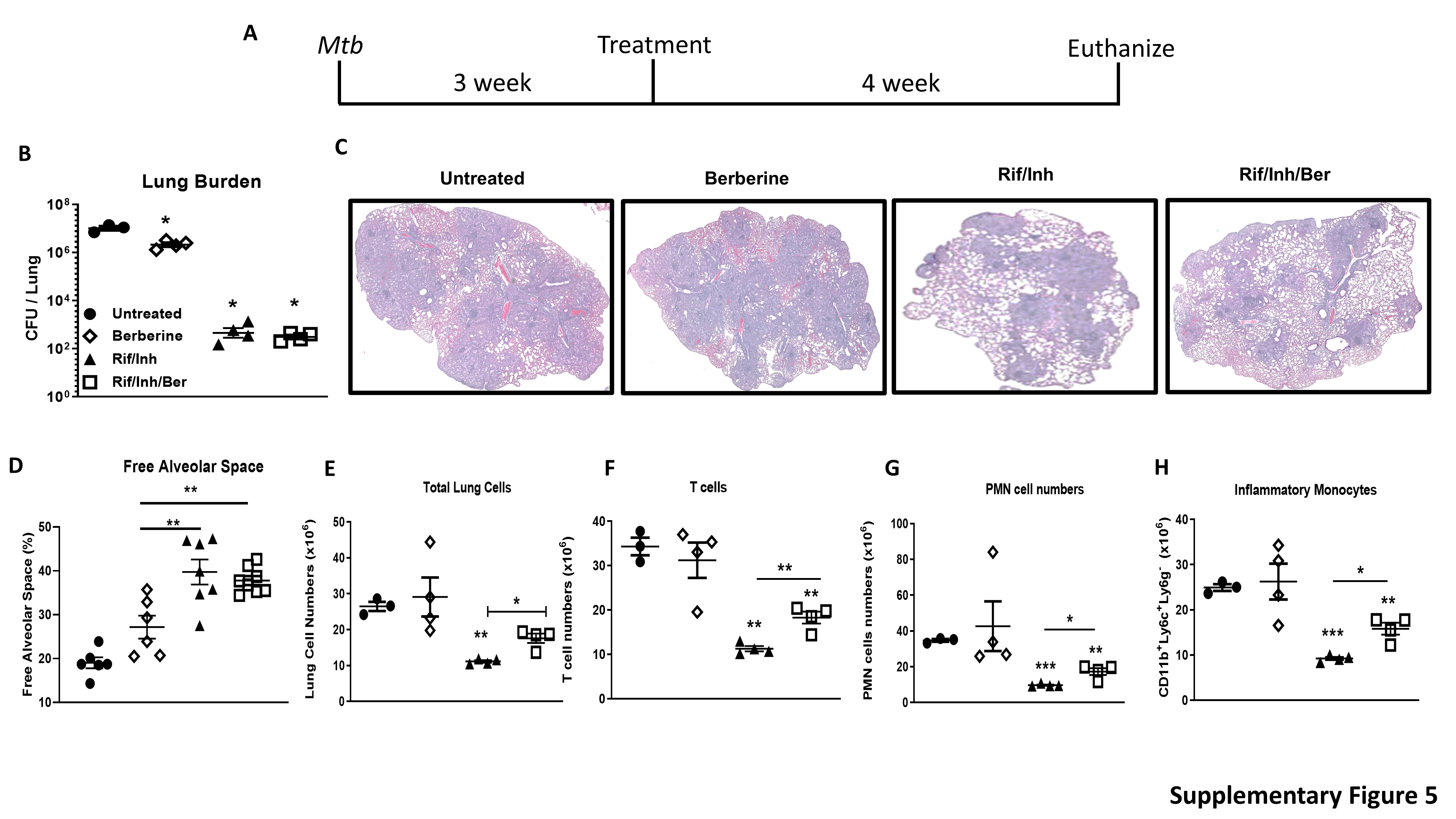

Supplement: Supplementary Figure 5 — Berberine as an adjunctive therapy against Mtb infection in C3HeB/FeJ (Kramnik) mice. (A) Mice were infected with Mtb Erdman (100 CFU) via aerosol inhalation. After 3 weeks of infection, mice were either left untreated or treated with berberine (1 mg/ml) or rifampin/isoniazid (0.1 mg/ml) and rifampin/isoniazid with berberine in drinking water for 4 weeks as shown in the layout. (B) Mycobacterial burdens in the lungs. (C) Representative lung section for pathology evaluated by H&E staining. (D) Quantification of free alveolar air spaces. Single-cell suspension of the lung was analyzed for immune cell populations by flow cytometry. (E) Total lung cells, (F) T cells (CD3+CD11b-Ly6G-Ly6C-), (G) polymorphonuclear cells (CD11b+Ly6G+Ly6C+) and (H) inflammatory monocytes (CD11b+Ly6G-Ly6C+) in the lungs at 4 weeks post-treatment. Data represented as mean ± SEM of n = 3-4 mice per group from one experiment, analysed by one-way ANOVA with Tukey post-hoc test defining differences in all groups as significant *P ≤ 0.05; **P ≤ 0.01; ***P ≤ 0.001. [file Image_5.tif]
